# Supplementary material for: Oxidative Stress and Emergence of Psychosis
Source: Antioxidants (Basel). 2022 Sep 21;11(10):1870. doi: 10.3390/antiox11101870 (PMC9598314; doi:10.3390/antiox11101870)
Supplement: Supplementary file 1 [file antioxidants-11-01870-s001.zip › antioxidants-1885667-supplementary.pdf]

Supplementary Table S1. Number of participants included in each study.

Study	Schizophrenia (n=)	Controls (n=)	First Episode Psychosis (n=)	Ultra High Risk (n=)	First Degree Relatives (n=)
[28]	60	30			33
[29]		29		13	
[30]	50	50			50
[31]		82			14
[32]		98	102		
[33]		40	23		
[34]	49	49	47		
[23]		31	27		
[35]		20	20		
[36]		28	28		
[37]	40	80	40		
[38]		152	354		
[39]		25	29		
[40]		37	50		
[41]		97	105		
[42]		65	149		
[43]	66	101			
[44]		43	22		
[45]	100	100	78		
[46]	38	40			
[47]	18	18			
[48]		38	38		
[49]	40	40			
[50]		68	60		
[51]		50	54		
[52]	30	16	22		
[53]		16	26		
[54]	14	26			
[55]	15	18			
[56]	25	20			
[57]	24	24			

[58]	53	36
[59]	100	51
[60]	40	35
[61]	48	32
[62]	75	30
[63]	31	27
[64]	50	50
[65]	41	43
[66]	46	50
[24]	189	60
[25]	189	60
[26]	164	50
[67]	92	50
[68]	73	73
[69]	30	30
[70]	85	75
[71]	52	48
[72]	46	50
[73]	41	43
[74]	29	25
[75]	20	20
[76]	48	40
[77]	17	15
[78]	12	6
[79]	35	35
[80]	60	40
[81]	45	34
[27]	296	181
